# Supplementary material for: A UMLS-based spell checker for natural language processing in vaccine safety
Source: BMC Med Inform Decis Mak. 2007 Feb 12;7:3. doi: 10.1186/1472-6947-7-3 (PMC1805499; doi:10.1186/1472-6947-7-3)
Supplement: Additional file 13 — RAP application directory. Sets up directory for RAP (RDF application for PHP) source code [file 1472-6947-7-3-S13.gz › rap/doc/phpdoc/model/Model.html]

xml version="1.0" encoding="iso-8859-1"?


Docs For Class Model


## Class Model

Description

Description |
Descendents
| Methods (details)

Abstract superclass of MemModel and DbModel. A model is a programming interface to an RDF graph.

An RDF graph is a directed labeled graph, as described in http://www.w3.org/TR/rdf-mt/. It can be defined as a set of <S, P, O> triples, where P is a uriref, S is either a uriref or a blank node, and O is either a uriref, a blank node, or a literal.

History:

- access: public
- author: Radoslaw Oldakowski <radol@gmx.de>
- author: Daniel Westphal <mail@d-westphal.de>
- version: V0.9.1

Located in /model/Model.php (line 36)

```
Object
   |
   --Model
```

Direct descendents

Description |
Descendents
| Methods (details)

| Class | Description |
| --- | --- |
| DbModel | This class provides methods for manipulating DbModels from DbStore. |
| MemModel | A MemModel is an RDF Model, which is stored in the main memory. |

Method Summary

Description |
Descendents |
Methods (details)

Model
Model
()

object MemModel
findForward
(object Node $subject, object Node $predicate, object MemModel $object, [mixed $newModel = NULL])

string
getBaseURI
()

object MemModel
&getMemModelByRDQL
(string $queryString, [boolean $closure = FALSE])

object OntModel
&getOntModel
(constant $vocabulary)

object MemModel
&getResmodel
(constant $modelType, string $baseURI)

void
load
(string $filename, [string $type = NULL], [boolean $stream = false])

string,
visualize
([string $format = "dot"], [boolean $short\_prefix = TRUE])

Methods

Description |
Descendents |
Methods (details)

Constructor Model (line 65)

Notice for people who are used to work with older versions of RAP.

- access: public
- throws: PHPError

Model

Model
()

findForward (line 267)

Searches for triples using find() and tracks forward blank nodes until the final objects in the retrieved subgraphs are all named resources.

The method calls itself recursivly until the result is complete. NULL input for subject, predicate or object will match anything. Inputparameters are ignored for recursivly found statements. Returns a new MemModel or adds (without checking for duplicates) the found statements to a given MemModel. Returns an empty MemModel, if nothing is found. !!!!!!!!!!!!!!!!!!!!!!!!!!!!!!!!!!!!!!!!!!!!!!!!!!!!!!!!!!!!!!!!!!!!!! WARNING: This method can be slow with large models. NOTE: Blank nodes are not renamed, they keep the same nodeIDs as in the queried model! !!!!!!!!!!!!!!!!!!!!!!!!!!!!!!!!!!!!!!!!!!!!!!!!!!!!!!!!!!!!!!!!!!!!!!

- throws: PhpError
- access: public
- author: Anton K�stlbacher <anton1@koestlbacher.de>

object MemModel

findForward
(object Node $subject, object Node $predicate, object MemModel $object, [mixed $newModel = NULL])

- object Node
  $subject
- object Node
  $predicate
- object MemModel
  $object

getBaseURI (line 80)

Return current baseURI.

- access: public

string

getBaseURI
()

getMemModelByRDQL (line 321)

Perform an RDQL query on this Model. Should work with all types of models.

This method returns a MemModel containing the result statements. If $closure is set to TRUE, the result will additionally contain statements found by the findForward-method for blank nodes. !!!!!!!!!!!!!!!!!!!!!!!!!!!!!!!!!!!!!!!!!!!!!!!!!!!!!!!!!!!!!!!!!!!!!! WARNING: If called with $closure = TRUE this method can be slow with large models. !!!!!!!!!!!!!!!!!!!!!!!!!!!!!!!!!!!!!!!!!!!!!!!!!!!!!!!!!!!!!!!!!!!!!!

- access: public
- author: Anton K�stlbacher <anton1@koestlbacher.de>
- author: code snippets taken from the RAP Netapi by Phil Dawes and Chris Bizer

object MemModel

&getMemModelByRDQL
(string $queryString, [boolean $closure = FALSE])

- string
  $queryString
- boolean
  $closure

getOntModel (line 234)

Returns an OntModel with this model as baseModel.

$vocabulary has to be one of the following constants (currently only one is supported): RDFS\_VOCABULARY to select a RDFS Vocabulary. You can supply a base URI

- access: public

object OntModel

&getOntModel
(constant $vocabulary)

- constant
  $vocabulary

getResmodel (line 218)

Returns a ResModel with this model as baseModel.

- access: public

object MemModel

&getResmodel
(constant $modelType, string $baseURI)

- constant
  $modelType
- string
  $baseURI

load (line 97)

Load a model from a file containing RDF, N3, N-Triples or a xhtml document containing RDF.

This function recognizes the suffix of the filename (.n3 or .rdf) and calls a suitable parser, if no $type is given as string ("rdf" "n3" "nt"); If the model is not empty, the contents of the file is added to this DbModel.

- access: public

void

load
(string $filename, [string $type = NULL], [boolean $stream = false])

- string
  $filename
- string
  $type
- boolean
  $stream

---

Redefined in descendants as:

- InfModelF::load()
  : Load a model from a file containing RDF, N3 or N-Triples.

visualize (line 436)

Alias for RDFUtil::visualiseGraph(&$model, $format, $short\_prefix)

!!!!!!!!!!!!!!!!!!!!!!!!!!!!!!!!!!!!!!!!!!!!!!!!!!!!!!!!!!!!!!!!!!!!!! Note: See RDFUtil for further Information. !!!!!!!!!!!!!!!!!!!!!!!!!!!!!!!!!!!!!!!!!!!!!!!!!!!!!!!!!!!!!!!!!!!!!!

- return: binary
- throws: PhpError
- access: public
- author: Anton K�stlbacher <anton1@koestlbacher.de>

string,

visualize
([string $format = "dot"], [boolean $short\_prefix = TRUE])

- string
  $format
- boolean
  $short\_prefix

#### Inherited Methods


Inherited From Object

> Object::toString()

Documentation generated on Thu, 7 Jul 2005 13:42:04 +0200 by phpDocumentor 1.3.0RC3
